# Rhombohedral Phase Formation in Yttria-Stabilized Zirconia Induced by Dental Technical Tools and Its Impact on Dental Applications

**DOI:** 10.3390/ma15134471

**Published:** 2022-06-24

**Authors:** Markus Wertz, Michael Benno Schmidt, Hieronymus Hoelzig, Maximilian Wagner, Bernd Abel, Gert Kloess, Sebastian Hahnel, Andreas Koenig

**Affiliations:** 1 Department of Prosthodontics and Material Sciences, Leipzig University, 04103 Leipzig, Germany; michael3.schmidt@klinik.uni-regensburg.de (M.B.S.); maximilian.wagner@iom-leipzig.de (M.W.); sebastian.hahnel@ukr.de (S.H.); 2 Department of Prosthetic Dentistry, University Hospital of Regensburg, 93053 Regensburg, Germany; 3 Institute of Mineralogy, Crystallography and Materials Science, Leipzig University, 04275 Leipzig, Germany; hieronymus.h@gmx.de (H.H.); kloess@uni-leipzig.de (G.K.); 4 Leibniz Institute of Surface Engineering (IOM), Leipzig University, 04318 Leipzig, Germany; 5 Wilhelm-Ostwald-Institute for Physical and Theoretical Chemistry, 04103 Leipzig, Germany; bernd.abel@iom-leipzig.de

**Keywords:** dental zirconia, X-ray diffraction, fixed dental prosthesis, computer-aided manufacturing, phase composition

## Abstract

In the study the influence of different dental technical tools on the surface temperature and phase composition of fixed dental prostheses (FDPs) made of yttria-partially stabilized zirconia polycrystals (3Y-/4Y-/5Y-PSZ) was investigated. FDPs were fabricated by using computer-aided manufacturing (CAM). The FDPs were treated with a contra-angle handpiece equipped with different burs and polishers. The resulting surface temperatures were measured with a thermographic camera, and the resulting phase transformations were investigated by X-ray diffraction and quantified by Rietveld refinement. Processing with burs resulted in no phase transformation, but a preferred orientation shift. Using coarse polisher induced a phase transformation to the rhombohedral phase, while fine polishers produced no relevant phase transformations and no preferred orientation shift. Compared to the monoclinic phase (ca. 9% theoretical volume increase), which is associated with low-temperature degradation (LTD), the rhombohedral phase is much more voluminous (ca. 15% theoretical volume increase) and distorted and, therefore, has a greater degradation potential.

## 1. Introduction

In recent years, tooth-coloured materials such as ceramics and resin composites have become very popular for application in monolithic aesthetic dental restorations [1,2,3,4,5]. As yttria-partly stabilized zirconia (Y-PSZ), compared to other dental materials, features outstanding mechanical performance [6] (flexural strength between 750 and 1300 MPa [7,8]) and its optical properties such as translucency [9] are almost similar to enamel. The material is biologically inert [8], has a high X-ray opacity, AND can be used for a broad variety of dental restorations, including fixed dental prostheses (FDPs), primary telescopic crowns, dental implants, and implant superstructures [4,5]. The survival rates are very high and similar to metal alloys [10]. They ranged for zirconia implants from about 76% (first generation [11]) to 95% after one to seven years [12], for fixed complete dentures (FCDs) 100% after one to seven years [5]), and for frameworks 100% after ten years [13].

The translucency of Y-PSZ increases with a reduction in the grain size of the pressed powder, the porosity, and the aluminium content, and with an increase in the yttria content [14]. For the question of indication, the yttrium oxide content in particular has proven to be a decisive material parameter, as it can be used to control the phase composition and thus, above all the optical and mechanical properties.

In general, Y-PSZ consists of a mixture of cubic (C; spacegroup Fm$\bar{3}$m) and tetragonal (T, T′, and T′′; spacegroup P4_2_/nmc) high-temperature phases, which are metastable at room temperature [15,16,17]. There are also several tetragonal phases T, T, and T′′ [16,17], two orthorhombic high-pressure phases (O and O’; Pbca and Pnam), and a monoclinic phase (M; spacegroup P2_1_/c), which is the only stable phase under standard conditions [15,16,17]. 

When subjected to thermal [18,19,20] or mechanical [21,22,23] stress, Y-PSZ may undergo a slow tetragonal (T) to monoclinic (M) transformation [24,25,26,27] under certain conditions, which is fostered by humidity [18,21]. 

Because of the resulting local volume expansion and the optical biaxiality, this aging phenomena (low temperature degradation; LTD) degrades the physical and optical properties of zirconia severely [15,18,19,21,28,29].

During cooling after sintering, the initially present tetragonal phase T′ separates into yttria-rich (C, T′′) and yttria-lean (M, T) phases [16,17,30]. Because of the low yttria solubility of the monoclinic phase, only the yttria-lean tetragonal phase T transforms in the monoclinic phase M, while the yttria-rich tetragonal phase T′′ and the cubic phase C are not transformable and, therefore, not subjected to low-temperature degradation [18,29].

Under mechanical loading (alumina particle blasting and grinding in the lab), the formation of a rhombohedral/trigonal phase (R; R$\bar{3}$) was observed [31,32,33,34]. This phase is a hettotype of the cubic phase (spacegroup Fm$\bar{3}$m → spacegroup R$\bar{3}$). It retains only the trigonal symmetry element $\bar{3}$ and features–compared to the cubic phase–a highly distorted elementary cell [33,35,36].

Prosthetic treatments include the examination of aesthetic and functional parameters such as the marginal fit between crown and tooth, proximal contact, and static and dynamic occlusion [37]. In many cases, occlusal adjustments are necessary, as interferences in the occlusion may cause problems with teeth and the temporomandibular joint, which may ultimately impair quality of life [38]. Occlusal adjustments are commonly performed with fine diamond burs, which are followed by a polishing regime. Adequate polishing is particularly relevant in restorations fabricated from zirconia, as insufficiently polished zirconia surfaces may cause increased abrasion in antagonistic natural tooth tissues [39,40].

It is well known that mechanical loading [8,32,41,42,43,44,45] of zirconia, e.g., as induced by polishing or grinding, may—under certain conditions—lead to a phase transformation. Song et al. [46] defined four different types of stresses that are applied on Y-PSZs during abrasion with a diamond bur and quantified them using finite element analysis (FEA). The various stress types include tensile (max. 904 MPa), shear (max. 588 MPa), compressive (max. 878 MPa), and theoretical von Mises (max. 885 MPa to 1974 MPa) stress [46]. Some of these stresses exceed the flexural strength of zirconia (cf. Table 1).

The study examined the influence of commonly used diamond burs and polishing equipment on the phase composition of fixed dental prostheses (FDPs) fabricated from 3Y-, 4Y-, and 5Y-PSZs. The boundary conditions necessary for the phase transformation (temperature distribution, mechanical effects) were determined experimentally. The results were compared to know phase transformation driven phenomena such as LTD. 

## 2. Materials and Methods

### 2.1. Materials and Sample Preparation

Six FDPs fabricated from Y-PSZs with different yttria contents supplied either by Dental Direkt (Dental Direkt GmbH, DE-32139 Sprenge; DD) or VITA Zahnfabrik (Vita Zahnfabrik H. Rauter GmbH & Co. KG, DE-79704 Bad Säckingen; VT) were used (Table 1). The FDPs were produced using computer-aided manufacturing (CAM) techniques in accordance with the instructions issued by manufacturers employing an inLab MC X5 (Dentsply Sitrona Deutschland GmbH, DE-64625 Bensheim) 5-axis milling machine and the CAD software Ceramill Mind 2.4 7437 (Amann Girrbach AG, AT-6842 Koblach) (cf. [47]).

**Table 1:** Overview of the Y-PSZs from two manufacturers used for producing FDPs.

Abbreviation	Product	LOT	Yttria Content mol % ^1^	Flexural Strength MPa ^1^
3Y_VT	VITA YZ HT	83,290	3	1100
3Y_DD	DD Bio ZX2	5,032,106,002	3	1250
4Y_VT	VITA YZ ST	65,890	4	>850
4Y_DD	DD cube ONE	7,162,042,001	4	>1250
5Y_VT	VITA YZ XT	61,962	5	>600
5Y_DD	DD cubeX^2^	8,032,028,002	5	800

^1^ According to the manufacturer.

The FDPs were processed from pre-sintered round discs (Ø 98.5 mm). For investigations, a premolar crown from the upper jaw (wall thickness: buccal/palatinal: 2.45 mm; mesial: 0.66 mm; distal: 0.69 mm) was manufactured. To simplify the X-ray diffraction (XRD) measurements, the FDPs featured flat occlusal surfaces without cusps. Sintering was conducted according to the instructions of the manufacturers at 1450 °C (3Y- and 5Y-PSZ) and 1530 °C (4Y-PSZ) using a zirconia sintering furnace (VITA Zyrcomat 6000 MS, Vita Zahnfabrik H. Rauter GmbH & Co. KG, Germany) (cf. [47]). For glace firing, glaze paste was prepared from VITA Akzent plus Glaze LT and VITA plus powder fluid. Heating was performed according to the instructions of the manufacturer with a vacuum furnace (VITA Vacumat 6000 M, Vita Zahnfabrik H. Rauter GmbH & Co. KG, DE-79704 Bad Säckingen, Germany) (cf. [47] Table 2). 

To simulate dental adjustments in occlusion, coarse (842KR) and fine (8837KR) diamond burs (both from Komet Dental, Lemgo, Germany) were used with an EXPERTtorque Mini LUX E677 L contra-angle handpiece (KaVo, Biberach an der Riß, Deutschland). Subsequently, polishing was simulated using typical coarse (Cera Glaze P3032A) and fine (Cera Glaze P30032A) polishers (both by NTI, Khala, Germany) (Table 3).

### 2.2. Methods

#### 2.2.1. Mechanical Loading

The manual mechanical vertical load, which was applied to the bur/polisher while processing was analysed using a universal testing machine (Retro line, ZwickRoell, Ulm, Germany). The force was applied directly on the measurement spot (Table 3).

#### 2.2.2. Thermographic Analysis

Processing with the burs and polishers was filmed using an MWIR A6700-InSb thermal imaging camera (Teledyne FLIR LLC, Wilsonville, Oregon, USA) with a thermal sensitivity of <18 mK. The dataset was analysed using ResearchIR 4.40.11 software (Teledyne FLIR LLC, Wilsonville, Oregon). The temperatures on the crowns were measured directly after removing the bur from the surface. For each measurement, two temperature ranges (77.4–216 and 146.6–323.6 °C for the burs and 20–98.1 °C and 146.6–216 °C for the polishers) were applied.

#### 2.2.3. X-ray Diffraction (XRD)

**Figure 1:**
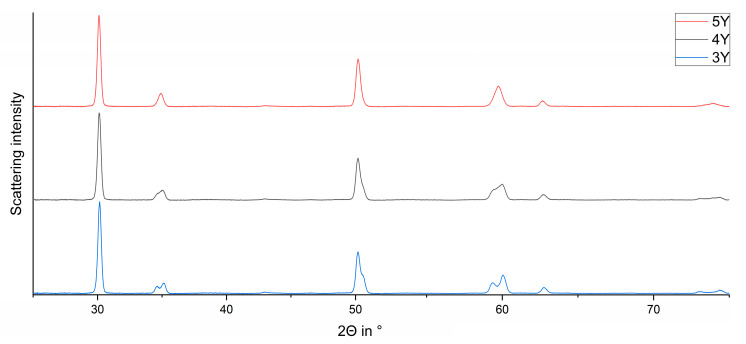
Depiction of the reference FDPs fabricated from 3Y-VT_1, 4Y-VT_1, and 5Y-VT_1.

The phase composition (Figure 1) was analysed using a D8 Discover (Bruker AXS Advanced X-ray Solutions GmbH, Karlsruhe, Germany) X-ray diffractometer with a VÅNTEC-500 (Vantec Thermal Technologies, Fremont, CA, USA) area detector. CuKα radiation (λ = 1.5418 Å) and X-ray settings of 40 kV and 40 mA were used. For gathering data, the measurement setup described by Wertz et al. [47] was applied. The integration was carried out with the software DIFFRAC.EVA (Version 3.1; Bruker AXS Advanced X-ray Solutions GmbH, Karlsruhe, Germany). 

#### 2.2.4. Rietveld Refinement

TOPAS 4.2 software (Bruker AXS Advanced X-ray Solutions GmbH, Karlsruhe, Germany) was used for Rietveld refinement. Structural data were derived from the literature [31,48,49] and adapted with data from various publications [15,16,17,41,50]. The XRD curve calculated includes structural models for the monoclinic (M), tetragonal (T, T′′), cubic (C), and rhombohedral/trigonal (R) [47].

**Figure 2:**
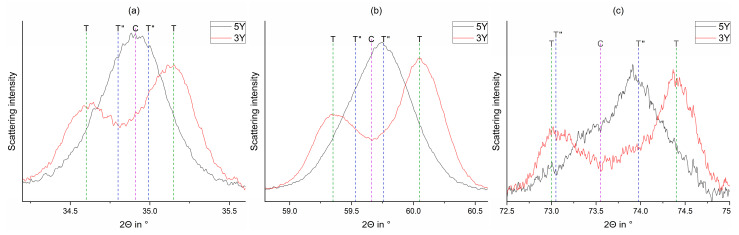
Comparison of the observed curves of the reference FDPs fabricated from 3Y_VT_1 (red) and 5Y_VT_1 (black) around 35° (**a**), 60° (**b**), and 74° (**c**) in 2Θ (from Figure 1) with their phase composition (T, T′´, C).

Important reflections at ~35°, ~60°, and ~74° in 2Θ (Figure 2a–c) were used for differentiating the tetragonal phase T from the tetragonal phase T′′ and the cubic phase C.

With increasing Yttria content, the tetragonal phase fraction T′′ increases at the expense of the tetragonal phase T. The corresponding reflections are a) 0 0 2_T/T′′_, 1 1 0 _T/T′′_ and 0 0 2_C_; b) 0 1 3_T/T′′_, 2 1 1 _T/T′′_ & 3 1 1_C,_ and c) 0 0 4_T/T′′_, 2 2 0 _T/T′′_, and 0 0 4_C_.

The increasing surface roughness induced by treatment with dental technical tools was compensated with a surface roughness correction [51]. Texture effects were refined using a preferred orientation approach according to March—Dollase [52,53]. The resulting refinement was improved using optical and numerical parameters in an iterative process. Diamond 4 software (Version 4.6.5, Crystal Impact GbR, Bonn, Germany) was used to display the phases and to determine parameters such as the number of atoms per unit cell.

## 3. Results

In general, identical phase compositions were found between the manufacturers for the processing steps and the different yttria contents. Therefore, only changes which were detected for both manufactures are presented in detail in the sections. The phase composition of all measurements after Rietveld refinement are deposited in the Appendix A (Table A1, Table A2, Table A3, Table A4, Table A5 and Table A6).

### 3.1. Coarse and Fine Diamond Burs

The usage of all coarse and fine diamond burs induced no phase transformation, but a preferred orientation shift from 1 1 0_T/T′′_ to 0 0 1_T/T′′_.

**Figure 3:**
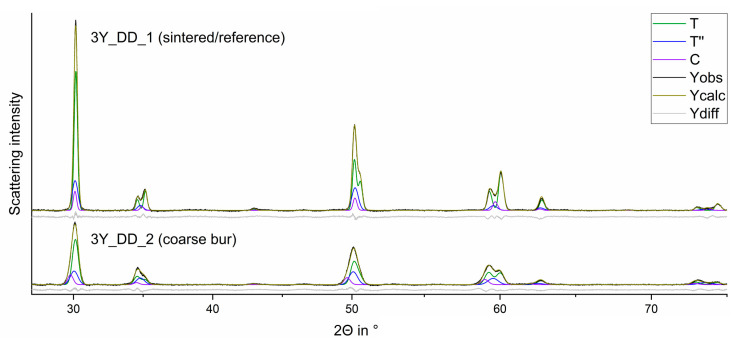
Observed (Yobs) and calculated (Ycalc) curves of FDPs 3Y_DD_1 (above; sintered FDP) and 3Y_DD_2 (below; coarse diamond bur) and the curves of the calculated phase fractions.

Figure 3 displays the diffractograms prior (a) and after (b) treatment with a coarse diamond bur. The main phases are the tetragonal phases T and T′′. 

The reflections at 35°, 60°, and 74° in 2Θ show a strengthening of 0 0 2_T/T′′_ (36°; left reflection), 0 1 3_T/T′′_ (60°; left reflection), and 0 0 4_T/T′′_ (60°; left reflection) for 1 1 0_T/T′′_ (34°; right reflection), 2 1 1_T/T′′_ (58°; right reflection), and 2 2 0_T/T′′_ (60°; right reflection).

**Figure 4:**
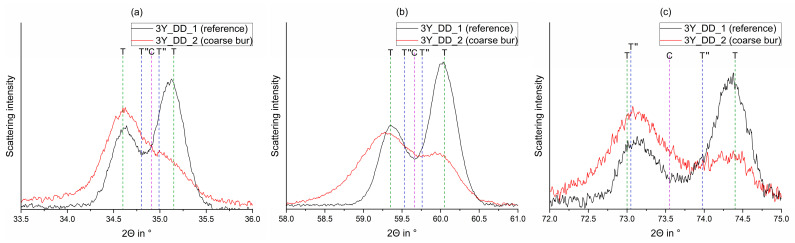
Comparison of the observed curves of the FDPs fabricated from 3Y_DD_1 (sintered, black) and 3Y_DD_2 (coarse diamond bur, red) around 35° (**a**), 60° (**b**), and 74° (**c**) in 2Θ (highlighted parts of Figure 3) with their phase composition (T, T´´, C).

A detailed look on certain areas (Figure 4) shows no substantial phase transformation (c.f. Table A1, Table A2, Table A3, Table A4, Table A5 and Table A6), so the scattering intensity shifts suggest a 0 0 1_T/T′′_ texture (preferred orientation in the grain) induced by the treatment with all coarse and fine burs. 

Processing with a coarse bur changes the ratio between 0 0 1 (left) and 1 1 0 (right) (preferred orientation change). The corresponding reflections are (a) 0 0 2_T/T′′_ (left), 1 1 0 _T/T′′_ (right), and 0 0 2_C_; (b) 0 1 3_T/T′′_ (left), 2 1 1 _T/T′′_ (right), and 3 1 1_C_; (c) 0 0 4_T/T′′_ (left), 2 2 0 _T/T′′_ (right), and 0 0 4_C_.

**Figure 5:**
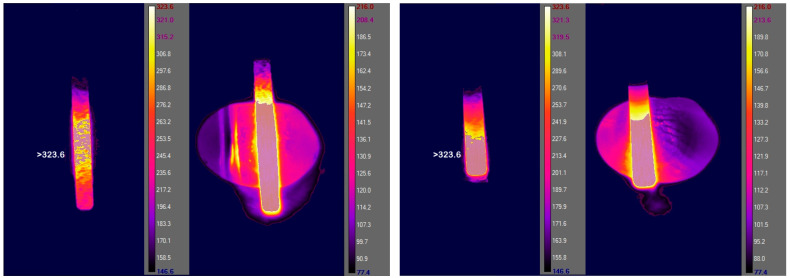
Thermograms of FDP surfaces processed with coarse (**left**, **middle-left**) and fine (**right**, **middle-right**) diamond burs. For each measurement two temperature ranges (77.4–216 and 146.6–323.6 °C) were applied.

Figure 5 depicts the temperature distribution of the coarse and fine burs during application. Both treatments produced very similar temperatures in the area of the surface contact point.

Comparing the temperature profile induced by treatment with the coarse and fine (Table 4) diamond burs, it is obvious that both treatments produced similarly high temperatures on the surfaces.

### 3.2. Coarse and Fine Polisher

**Figure 6:**
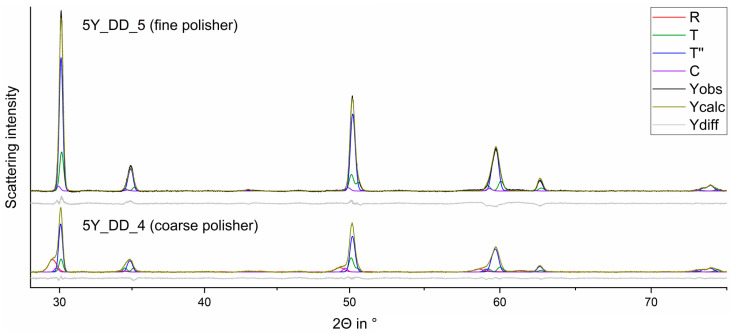
Observed (Yobs) and calculated (Ycalc) curves for FDPs fabricated from 5Y_DD_4 (above; coarse polisher) and 5Y_DD_5 (below; fine polisher) and the curves of the calculated phase fractions.

When coarse polishers (Figure 6) were applied on all samples listed in Table 1, substantial formation of a rhombohedral (trigonal R) phase was induced. The fine polisher induced no relevant phase transformation (c.f. Table A1, Table A2, Table A3, Table A4, Table A5 and Table A6).

**Figure 7:**
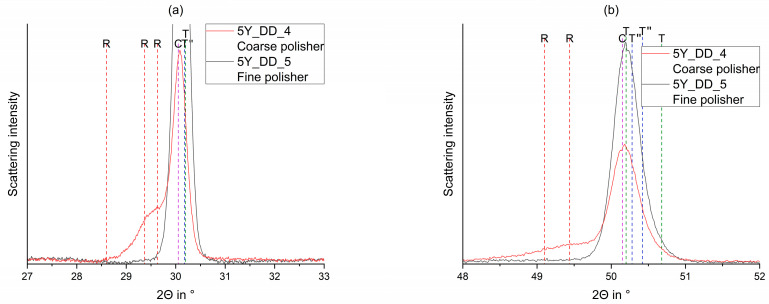
Comparison of the observed curves of the FDPs fabricated from 3Y_DD_4 (sintered, fine polisher; red) and 3Y_DD_5 (sintered, fine polisher; black) around 30° (**a**) and 50° (**b**) in 2Θ (highlighted parts of Figure 6).

Figure 7 shows the detailed indication of some rhombohedral reflections (R) exemplarily.

**Figure 8:**
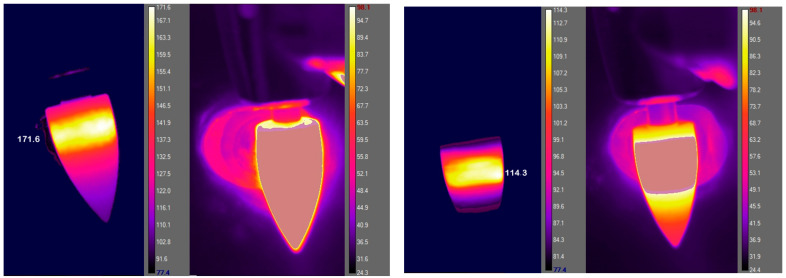
Thermograms of FDP surfaces processed with coarse (left, middle-left) and fine (right, middle-right) diamond burs. For each measurement two temperature ranges (20–98.1 and 146.6–216 °C) were applied.

The thermograms (Figure 8) gathered during treatments with coarse and fine polishers showed large temperature differences between the two treatments.

The temperatures observed on the surface of the FDPs and polishers were much higher during treatment with the coarse polisher than with the fine polisher (Table 5).

## 4. Discussion

Following the arguments of Wertz et al. [47], we have used a phase model for Rietveld refinement, which includes the two tetragonal phases T and T′′ as well as cubic phase C, monoclinic phase M, and trigonal/rhombohedral phase R. In regard to the tetragonal phases, the authors showed that including both phases substantially improves the refinement.

### 4.1. Coarse and Fine Diamond Burs

The usage of diamond burs leads to a preferred orientation shift, but no phase transformation. In the current study, the fine polisher induced no such preferred orientation shift, probably because of its finer surface structure.

**Figure 9:**
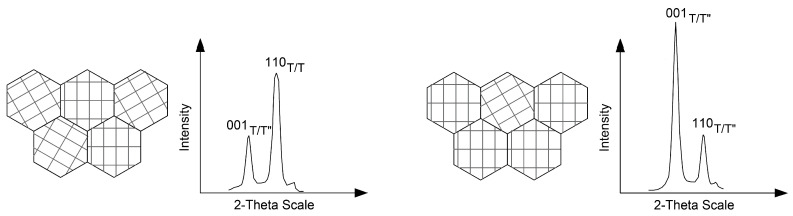
Schematic idealized illustration of grains with a preferred orientation of crystal structure and their impact on a diffractogram. The initially randomly oriented grains (**left**) are oriented towards 0 0 1_T/T′′_ (**right**). Since parts of the grains still have a preferred orientation towards 1 1 0_T/T′′_, a 1 1 0_T/T′′_ reflection remains. It should be noted that reflections 1 1 0_T/T′′_ and 0 0 1_T/T′′_ are located in two different spatial planes, and therefore, cannot be represented in a 2D drawing.

Since one stability mechanism in tetragonal zirconia includes a layered structure with strong (2.09–2.10 Å) Zr-O_1_ bonds within and weak (2.33–2.35 Å) Zr-O_2_ bonds between the layers [26,54], a preferred orientation shift (Figure 3 and Figure 4) through coarse or fine diamond burs may change this layered structure (Figure 9). 

### 4.2. Coarse and Fine Polisher

The coarse polisher induced the formation of a rhombohedral phase (R). Because of its metastability, the rhombohedral phase of zirconia has only rarely been reported. While it has been discussed [31,32,47,55] in other studies [20,56], typical reflections for the rhombohedral phase (R) were measured but not discussed.

**Figure 10:**
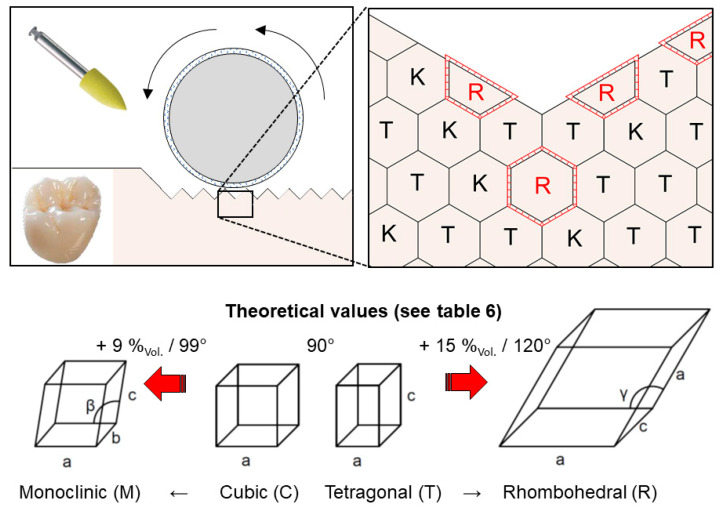
Schematic and idealized illustration of the formation of the rhombohedral phase and its consequences at the grain and unit cell levels. Polishing with coarse polishers induced a phase transformation to the rhombohedral phase (**above**). In comparison to the transformation to the monoclinic phase, a higher volume expansion (values based on atoms per volume ratio; cf. Table 6) and shear occur (**below**).

The elementary cell (Table 6 and Figure 10) of the rhombohedral phase (R) features a 15% higher volume per atom ratio than the cubic (C) and tetragonal phases (T, T′′) and a shear of 30° (cf. monoclinic elementary cell: 4–5% [15] to 9% higher; 9° shear [15]). Additionally, in the rhombohedral phase, every zirconium atom is bonded with only six oxygen atoms instead of eight atoms, as in the cubic and tetragonal phases (monoclinic phase: seven O- bondings per Zr- atom).

When the FDP is heated after polishing in dental practice (e.g., for glace firing), a retransformation from the rhombohedral to the tetragonal phase would be possible, which may cause shrinkage and microstrain formation.

These effects may be stronger than the effects of the LTD-related monoclinic phase transformation because of the stronger distortion (Table 6), the higher volume expansion, and the larger shear of the rhombohedral phase.

Kitano et al. [31] indicated that a rhombohedral to monoclinic phase transformation may also be operative. Therefore, the rhombohedral phase may also be subjected to processes such as low-temperature degradation (LTD) and potentially promote them.

Only treatment with the coarse polisher resulted in the formation of the rhombohedral phase. Subsequent firing (e.g., glace firing or regeneration firing) could retransform the rhombohedral phase into a tetragonal or cubic phase, which may cause additional stresses by shrinkage phenomena. The different results can be explained by the higher temperatures induced by treatment with the coarse polisher (Table 5) and the entry of shear forces [46,57].

The current study is limited by the low thickness of the surface, which can be analysed by X-ray diffraction (XRD), the harsh loading conditions, and the limited number of dental technical tools (Table 3) we used. Therefore, the practical consequences on this the laboratory study must determine in further investigations.

## 5. Conclusions

Our experiments within the limitations of the study show some conditions of the formation of a rhombohedral phase fraction (15–30%) of 3/4/5Y-PSZ through polishing with dental handpieces.

1. Polishing with coarse polishers can induce a partial rhombohedral phase transformation.
2. All diamond burs used induce a break up of the layer structure of Y-PSZ and a subsequent preferred orientation shift to 0 0 1 _T/T′′_ at the expense of 1 1 0 _T/T′′,_ but no phase transformation.
3. Treatment with fine polishers did not induce any relevant phase transformation.

Similar to the tetragonal to monoclinic phase transformation, the rhombohedral phase transformation induced a volume expansion (15% higher volume per atom ratio than the cubic or tetragonal phases) and shear (30°). Since only the coarse polisher induced this phase transformation, this indicates a metastable phase stability field, in which this phase transformation occurs. 

Further studies should address the conditions and prevention of the formation of the rhombohedral phase, especially the combination of different polishers in practically used polishing sets. It is necessary to clarify the practical impact of the above observations on the material properties (mechanical and optical) and clinical performance of FDPs fabricated from Y-PSZs. Additionally, the stability of the rhombohedral phase against subsequent firings and low-temperature degradation should be investigated.

## Figures and Tables

**Table 2:** Every different step of processing of FDPs. *Each* technical tool was applied individually to one sintered FDP.

Treatment Number	Processing Step
1	Sintered (glazed) FDP
2	Sintered (glazed) FDP processed with a coarse diamond bur
3	Sintered (glazed) FDP processed with a fine diamond bur
4	Sintered (glazed) FDP processed with a coarse polisher
5	Sintered (glazed) FDP processed with a fine polisher

**Table 3:** Overview of the dental technical tool used for processing FDPs.

Dental Technical Tool	Revolutions rpm	Vertical Load N	Time of Processing min
Coarse diamond bur	400,000	5–15	4
Fine diamond bur	400,000	5–15	4
Coarse polisher	10,000	5–15	4
Fine polisher	5000	5–15	4

**Table 4:** Maximum temperatures measured on the diamond bur and FDP surfaces.

	Max. Temperatures on the Diamond Burs in °C	Max. Temperatures on the FDPs in °C
Coarse diamond bur	>320	~190
Fine diamond bur	>320	~190

**Table 5:** Maximum temperatures measured on the polisher and FDP surfaces.

	Max. Temperatures on the Polisher in °C	Max. Temperatures on the FDP in °C
Coarse polisher	~175	~90
Fine polisher	~115	~65

**Table 6:** Properties of the different elementary cells of the refined phases.

Crystal System	O- Bonds per Zr- Atom	Atoms per Elementary Cell	Volume in Å^3^	Volume per Atom in Å^3^
Cubic	8	12	133	11
Tetragonal	8	6	67	11
Monoclinic	7	12	144	12
Rhombohedral	6	57	748	13

## Appendix A

**Table A1:** Quantification of the phase content of the FDPs fabricated from 3Y_VT. VT: Vita Zahnfabrik.

3Y_VT	M	R	T	T′′	C
3Y_VT_1 (as sintered)	0	0	82	7	11
3Y_VT_2 (coarse bur)	0	0	64	22	12
3Y_VT_3 (fine bur)	0	0	60	29	11
3Y_VT_4 (coarse polisher)	0	30	39	16	15
3Y_VT_5 (fine polisher)	0	0	74	8	18

**Table A2:** Quantification of the phase content of the FDPs fabricated from 3Y_DD. DD: Dental Direct.

3Y_DD	M	R	T	T′′	C
3Y_DD_1 (as sintered)	0	0	80	8	12
3Y_DD_2 (coarse bur)	0	0	67	17	16
3Y_DD_3 (fine bur)	0	0	69	20	11
3Y_DD_4 (coarse polisher)	0	14	68	14	4
3Y_DD_5 (fine polisher)	0	0	84	7	9

**Table A3:** Quantification of the phase content of the FDPs fabricated from 4Y_VT. VT: Vita Zahnfabrik.

4Y_VT	M	R	T	T′′	C
4Y_VT_1 (as sintered)	0	0	60	21	19
4Y_VT_2 (coarse bur)	0	0	44	39	17
4Y_VT_3 (fine bur)	0	0	42	47	12
4Y_VT_4 (coarse polisher)	0	30	25	34	11
4Y_VT_5 (fine polisher)	0	0	54	27	19

**Table A4:** Quantification of the phase content of the FDPs fabricated from 4Y_DD. DD: Dental Direct.

4Y_DD	M	R	T	T′′	C
4Y_DD_1 (as sintered)	0	0	60	24	16
4Y_DD_2 (coarse bur)	0	0	53	32	15
4Y_DD_3 (fine bur)	0	0	52	37	11
4Y_DD_4 (coarse polisher)	0	18	53	23	6
4Y_DD_5 (fine polisher)	0	0	60	29	11

**Table A5:** Quantification of the phase content of the FDPs fabricated from 5Y_VT. VT: Vita Zahnfabrik.

5Y_VT	M	R	T	T′′	C
5Y_VT_1 (as sintered)	0	0	24	64	12
5Y_VT_2 (coarse bur)	0	0	28	56	16
5Y_VT_3 (fine bur)	0	0	30	59	11
5Y_VT_4 (coarse polisher)	0	15	18	62	5
5Y_VT_5 (fine polisher)	0	0	28	57	15

**Table A6:** Quantification of the phase content of the FDPs fabricated from 5Y_DD. DD: Dental Direct.

5Y_DD	M	R	T	T′′	C
5Y_DD_1 (as sintered)	0	0	24	66	10
5Y_DD_2 (coarse bur)	0	0	23	57	20
5Y_DD_3 (fine bur)	0	0	22	61	17
5Y_DD_4 (coarse polisher)	0	18	15	60	7
5Y_DD_5 (fine polisher)	0	0	26	63	11
